# LONGITUDINAL CHANGES IN ALCOHOL USE ACROSS 20 YEARS: A DAILY DIARY STUDY

**DOI:** 10.1093/geroni/igae098.3398

**Published:** 2024-12-31

**Authors:** Sara Miller, David Almeida

**Affiliations:** The Pennsylvania State University, Pennsylvania, United States; Pennsylvania State University, University Park, Pennsylvania, United States

## Abstract

Longitudinal studies often identify declines in alcohol consumption with aging. However, drinking trajectories may vary across alcohol use indicators and person-level characteristics. The current study utilized three bursts of daily diary surveys from the National Study of Daily Experiences to examine changes in alcohol consumption across 20 years among a sample of adults aged 24 to 81. Participants (N=1,429; Mage=49.9) completed 2+ bursts of 8-day telephone diary interviews. Outcome measures included burst-level abstaining, drinking frequency, frequency of exceeding guidelines of the National Institute on Alcohol Abuse and Alcoholism (1+/2+ drinks for females/males), and average drinking quantity (i.e., number of drinks on drinking days). Moderating variables included baseline age, biological sex (1=Male), education (1=Less than high school to 5=Graduate school), and perceived physical health (1=Poor to 5=Excellent). Multilevel linear and logistic regression analyses revealed that alcohol use quantity declined over time, while frequency of drinking, frequency of exceeding guidelines, and likelihood of alcohol abstention were stable, adjusting for baseline sociodemographic, health behavior, psychosocial, and health characteristics. There were also individual differences in drinking trajectories. Older age was associated with drinking more frequently and consuming fewer drinks per occasion at baseline as well as stronger declines in most alcohol use indicators across time. Furthermore, graduate education was linked with increasing drinking frequency, while male sex and poorer physical health were associated with stronger declines in drinking quantity. Future studies should evaluate mechanisms of age-related declines in alcohol use and examine contributing factors of individual differences in alcohol use trajectories across adulthood.

